# Longitudinal characterisation of a model of chronic allergic lung inflammation in mice using imaging, functional and immunological methods

**DOI:** 10.1186/1476-9255-10-S1-P4

**Published:** 2013-08-14

**Authors:** Kumar Changani, Catherine Pereira, Simon Young, Robert Shaw, Simon P Campbell, Kashmira Pindoria, Steve Jordan, Katherine Wiley, Sarah Bolton, Tony Nials, Michael Haase, Mike Pedrick, Richard Knowles

**Affiliations:** 1 Respiratory Therapeutic Area, GlaxoSmithKline, Gunnels Wood Road, Stevenage, Hertfordshire, SG1 2NY, UK; 2 Platform Technology and Science, GlaxoSmithKline, Gunnels Wood Road, Stevenage, Hertfordshire, SG1 2NY, UK; 3 AstraZeneca, Alderley Park, Macclesfield SK10 4TG, UK; 4 Arachos Pharma, Stevenage Bioscience Catalyst, Gunnels Wood Road, Stevenage, Hertfordshire, SG1 2FX, UK; 5 Huntingdon Life Sciences, Woolley Road, Huntingdon, Cambridgeshire, PE28 4HS

## 

**Figure 1:**
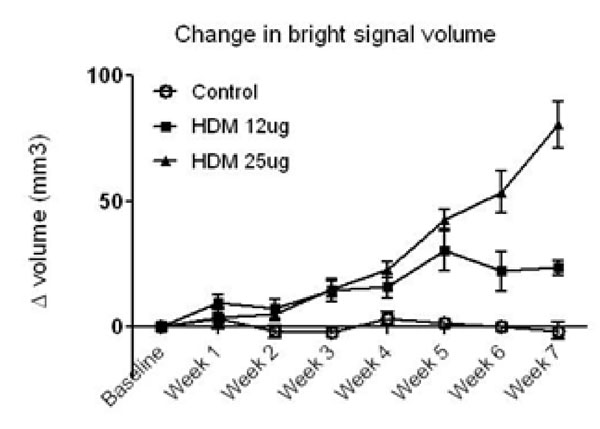
MRI data

**Figure 2:**
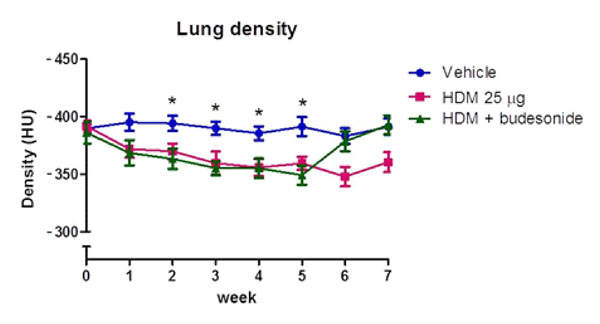
CT data

The present study investigated the role that imaging could have for assessing lung inflammation in a mouse model of a house dust mite (HDM) provoked allergic inflammation. Inflammation is usually assessed using terminal procedures such as bronchoalveolar lavage (BAL) and histopathology; however, magnetic resonance imaging (MRI) and computed tomography (CT) methods have the potential to allow longitudinal, repeated study of individual animals. Female BALB/c mice were administered daily either saline, or a solution of mixed HDM proteins sufficient to deliver a dose of 12µg or 25µg total HDM protein ± budesonide (1mg/kg, during weeks 5-7) for 7 weeks. Airway hyper- responsiveness (AHR) and IgE measurements were taken on weeks 3, 5 and 7. Following the last imaging session BALs were taken and lungs prepared for histology. MRI showed a gradual weekly increase in lung tissue intensity (LTI) in animals treated with HDM compared to control. The 25ug HDM group showed a continual significant increase in LTI between weeks 3-7, the 12ug HDM treated group showed similar rates of increase, and plateaued by week 5 (Figure 1). A corresponding increase in AHR, cell counts and IgE were observed. CT showed significant increases in lung tissue density from week 1 of HDM and this was maintained throughout the 7 weeks. Budesonide treatment reversed the increase in tissue density (Figure 2). MRI and CT therefore provide non-invasive sensitive methods for longitudinally assessing lung inflammation. Lung tissue changes could be compared directly with the classical functional and inflammatory readouts allowing more accurate assessments to be made within each animal and provide a clinically translatable approach.

